# Evaluation of absolute quantitation by nonlinear regression in probe-based real-time PCR

**DOI:** 10.1186/1471-2105-7-107

**Published:** 2006-03-03

**Authors:** Rasmus Goll, Trine Olsen, Guanglin Cui, Jon Florholmen

**Affiliations:** 1 Institute of Clinical Medicine, University of Tromso, Tromso, Norway; 2 Department of gastroenterology, University hospital of Northern Norway, Tromso, Norway

## Abstract

**Background:**

In real-time PCR data analysis, the cycle threshold (CT) method is currently the gold standard. This method is based on an assumption of equal PCR efficiency in all reactions, and precision may suffer if this condition is not met. Nonlinear regression analysis (NLR) or curve fitting has therefore been suggested as an alternative to the cycle threshold method for absolute quantitation. The advantages of NLR are that the individual sample efficiency is simulated by the model and that absolute quantitation is possible without a standard curve, releasing reaction wells for unknown samples. However, the calculation method has not been evaluated systematically and has not previously been applied to a TaqMan platform. *Aim: *To develop and evaluate an automated NLR algorithm capable of generating batch production regression analysis.

**Results:**

Total RNA samples extracted from human gastric mucosa were reverse transcribed and analysed for TNFA, IL18 and ACTB by TaqMan real-time PCR. Fluorescence data were analysed by the regular CT method with a standard curve, and by NLR with a positive control for conversion of fluorescence intensity to copy number, and for this purpose an automated algorithm was written in SPSS syntax. Eleven separate regression models were tested, and the output data was subjected to Altman-Bland analysis. The Altman-Bland analysis showed that the best regression model yielded quantitative data with an intra-assay variation of 58% vs. 24% for the CT derived copy numbers, and with a mean inter-method deviation of × 0.8.

**Conclusion:**

NLR can be automated for batch production analysis, but the CT method is more precise for absolute quantitation in the present setting. The observed inter-method deviation is an indication that assessment of the fluorescence conversion factor used in the regression method can be improved. However, the versatility depends on the level of precision required, and in some settings the increased cost effectiveness of NLR may justify the lower precision.

## Background

The use of real-time PCR in functional genomics has increased dramatically during the past decade. With this method, the detection of template accumulation in the PCR reaction is based on a fluorescent probe, or a fluorescent dye. The advantages compared to former PCR approaches are many: A: A closed compartment method decreases risk of contamination, as no post-PCR handling is necessary. B: The data used for calculation of quantity are collected as the PCR reaction runs, reducing the time span from pre-PCR procedures to final results are available. C: Compared to endpoint analyses of PCR reactions, real-time PCR is unmatched in precision – and D: An extreme dynamic range of 7–8 log_10 _[1,2].

In the software currently available, analysis of real-time data is generally based on the "cycle-threshold" (CT) method. Some packages offer curve-smoothing and normalisation, but the basic CT algorithm remains unchanged. Threshold fluorescence is calculated from the initial cycles, and in each reaction the CT value is defined by the fractional cycle at which the fluorescence intensity equals the threshold fluorescence. A standard curve can be used for absolute quantitation, or the comparative CT method can be used for relative quantitation [3].

The CT method is quite stable and straightforward, so why try to complicate things? The answer is that the precision of estimates is impaired if efficiency is not equal in all reactions. Uniform reaction efficiency is the most important assumption of the CT method. The simplest estimate of individual sample efficiency is calculated from the slope of the first part of the log-linear phase [4], and can be used for identification of outliers or correction of values from individual samples. The sigmoid curve fit or non-linear regression (NLR) [5], on the other hand, assumes a dynamic change in efficiency and closely resembles the observed course of fluorescence accumulation during the whole reaction. A further advantage of regression analysis is the possibility to generate estimates of initial copy number directly from the regression estimates, eliminating the need for a standard curve [6]. In small study series, the standard curve may be the best choice – but in a high-throughput production lab, elimination of the standard curve could liberate time and resources.

The first obstacle to the use of NLR is that the algorithm needs to be automated. The second and more important obstacle is that proper evaluation is missing both of the comparison of NLR with the CT method, and of the performance of NLR with probe-based chemistry. We therefore decided to develop and evaluate an automated regression model, to test if NLR is a real alternative to the traditional CT method.

## Results

**Figure 1:**
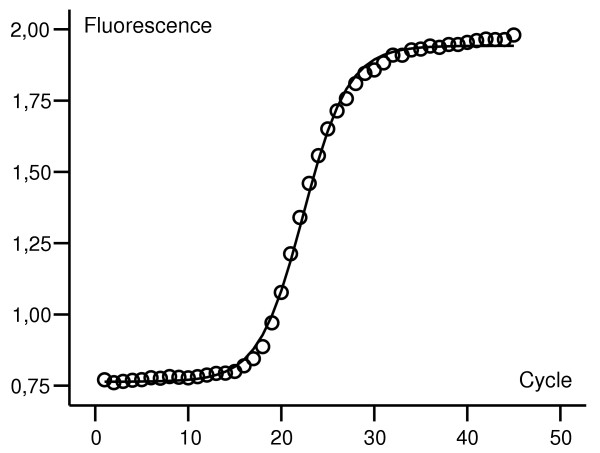
**Sigmoidal curve fitting. **A sigmoidal curve fit of fluorescence data from a real-time experiment (ACTB). Observed fluorescence plotted as data points (o), predicted fluorescence shown as a curve. The data set in this example is not background corrected.

**Figure 2:**
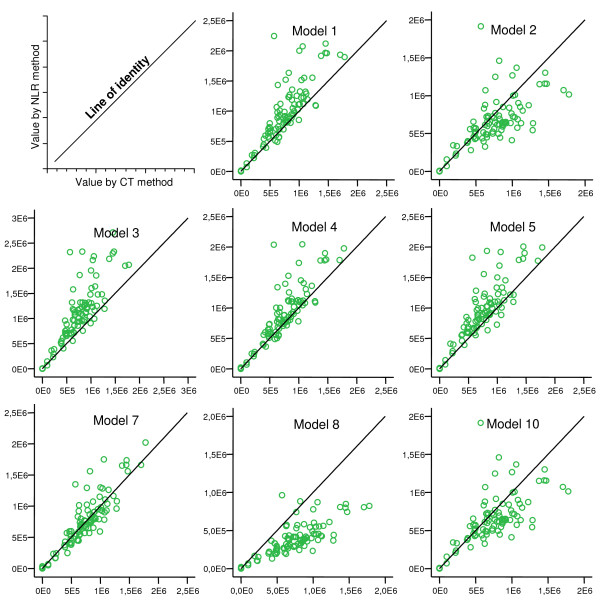
**Plots of copy numbers by NLR vs. CT. **ACTB mRNA measured by real-time PCR on extracts from human gastric mucosa. Plots show values (copies/μL) derived by NLR (Y-axis) vs. by CT (X-axis). A line of identity is inserted in each plot. Models that produced one or more bad fits are not shown.

Figure 1 shows an example of a curve-fit generated by NLR. In models 6, 9 and 11, one or more regressions returned bad fits (defined as generation of "impossible values" such as negative F_*max*, _etc.). In figure 2, plots of NLR- vs. CT-generated data are shown. Most models show a fair correlation. Models 3, 8, and 10 have higher bias than the rest, and the error is higher in models 2, 8, and 10. Models with one or more "bad fits" are not shown.

**Figure 3:**
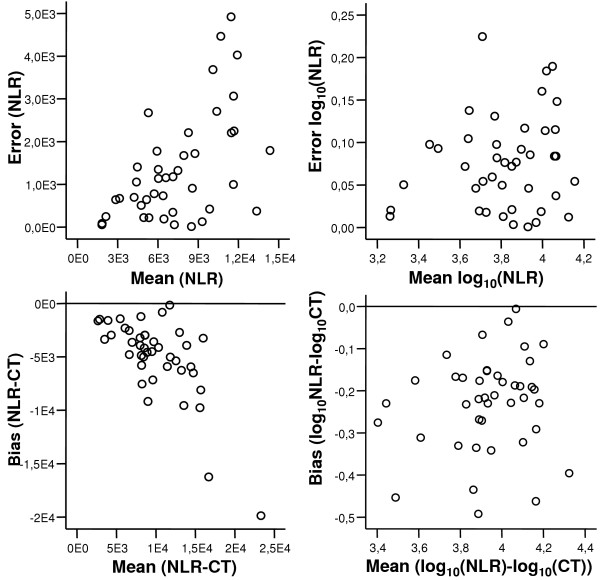
**Error vs. mean shows independence following log_10 _transformation. **Data generated from real-time RT-PCR assay for IL18 on RNA extracts from human gastric mucosa. Upper left plot shows error (numerical difference between duplicate values) vs. mean (of duplicate values) for values derived by the NLR method. As the error tends to increase with the mean, a plot based on log_10 _transformed copy numbers is shown (upper right), and independence can be observed. The same pattern was observed for the bias (difference between NLR- and CT-generated values), in the lower left plot. Again, a log_10 _transformation of the copy number values (lower right) can remove the trend, and independence can be observed.

**Figure 4:**
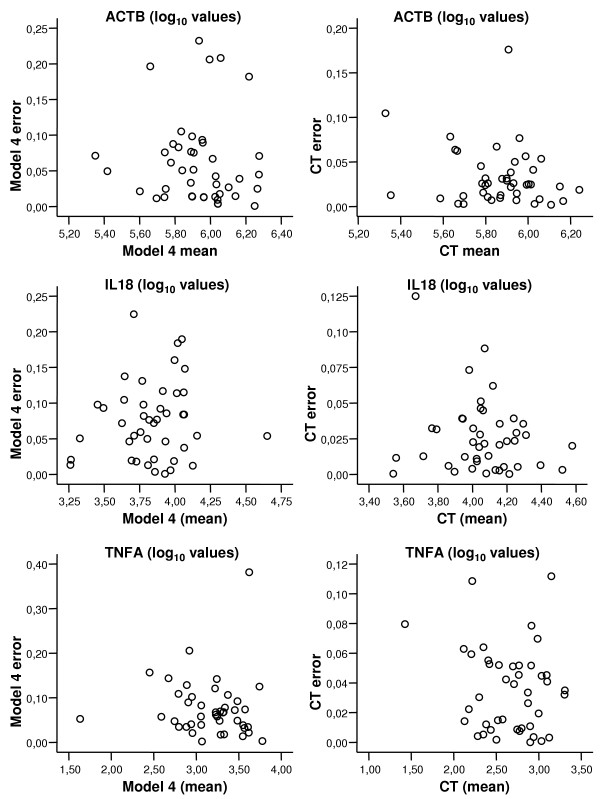
**Intra-assay variation by the NLR (model 4) and by the CT method. **Real-time RT-PCR data for ACTB, IL18 and TNFA on RNA extracts from human gastric mucosa. Plots show intra-assay variation or error (difference between duplicate values) vs. mean (of duplicates) calculated from log_10 _transformed copy number values. The left column is NLR data from model 4, the right column is CT-derived values. The errors observed seem to be independent of mean values. A 95 percentile of these data can then be calculated for evaluation of assay performance. Generally, the intra-assay variation is lower in CT-method derived values.

Altman-Bland plots were made of the numerical differences between duplicates (error) vs. duplicate means for each dataset and each regression model and the CT method. These plots showed an increase of error with increasing mean (an example of this is shown in figure 3). However, a log_10 _transformation of all final estimated values could resolve this pattern, and the error plots showed independence (figure 4 compares intra-assay variation with model 4 and with the CT method – for all assays). The intra-assay variation could then be characterised by a 95 percentile of the observed errors. The inverse log_10 _of this percentile can be interpreted as factor variation and recalculated to a percentage, as presented in table 2.

**Figure 5:**
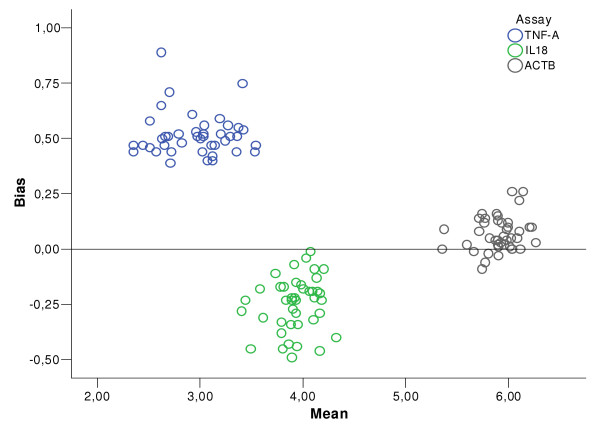
**Bias between the NLR and the CT method. **Real-time RT-PCR data for ACTB, IL18 and TNFA on RNA extracts from human gastric mucosa – a graphical representation of the observed bias between NLR and CT methods (inter-method agreement or inter-assay variation) in all three assays tested in this study. The final estimated copy numbers were transformed by log_10 _before calculation of bias. For these methods to be freely interchangeable, the bias plots should form a cloud around the zero line.

The mean copy number of duplicates was then analysed in plots of differences between NLR- and CT-derived values (bias) vs. means (of NLR- and CT-derived values). Again, independence could be observed after log_10 _transformation of the copy number values, but not in the raw data. In each experiment there was a relative bias, but when comparing the different experiments the bias was clearly not systematic. In figure 5, the bias of model 4 is shown in an Altman Bland plot containing data from all three experiments. The distribution of the data clouds indicates that each conversion factor varies between experiments in a random manner.

The calculated conversion factors ranged from 7.96E+10 to 3.07E+11 copies/fluorescence unit. Table 2 offers an overview of all models tested and key figures of their performance. The error percentiles stated are calculated on pooled data from all 3 assays, and the bias values are means of pooled numerical bias. As can be seen in figure 5, a simple average of pooled values would yield an erroneously low estimate of the bias, so the overall bias of each regression model has been calculated as an average of numerical bias values. For evaluation of the modifications applied, table 3 offers an overview of resulting R^2 ^mean, error, and bias changes.

## Discussion

### CT method

In the CT method equal efficiency in all reactions is assumed, and the impact of this assumption on final estimates has been underlined previously [4,7,8]. Tichopad [4] presented a standardised, automatable algorithm for estimation of sample specific efficiency, and a similar approach was published by Ramakers et al [8]. These models calculate efficiency at the early log-linear phase, and assume homogenous efficiency before that. However, calculation of sample specific efficiency was also evaluated by Peirson et al [9], who concluded that this approach was good for detection of outliers, but individual efficiency correction did not improve the precision of absolute quantitation.

The CT method has also been combined with curve-smoothing to obtain background correction and data smoothing (in the soFAR software package [10] and by Larionov et al [11], who also included amplitude normalisation). The latter approach may produce nice curves, but especially amplitude normalisation will change the slope of the log-linear phase and thereby mask differences in reaction efficiency.

### NLR

Theoretically, a calculation of template accumulation that mimics the dynamic change in PCR efficiency, and includes a larger array of the collected fluorescence data, could be more precise than the CT method. Alternatives to CT-based calculation have been suggested previously [5,6,12,13]. One model that assumes a dynamic change in efficiency is the sigmoidal curve fit [5], though limitations apply [6,12]. Especially the late plateau phase of the reaction is difficult to fit in this mathematical model. Rutledge suggested removal of observations from the late plateau phase to increase goodness-of-fit to the remaining data. Principal objections aside, the latter approach is less well suited for automation. To solve this problem we tested weighted analysis, which performed well in automation but unfortunately did not improve the precision of estimates.

### Automation

Algorithms for this type of analysis should be independent on user input apart from the raw data, to eliminate user-dependent bias. In general, "mass production" techniques should be used with caution in complicated regression models, as small errors may impair the precision of the final estimates [14]. Of the models initially investigated in this study, three produced one or more bad fits when automated – which illustrates a potential disadvantage of NLR when compared to CT analysis. The remaining eight models seemed robust, and could be evaluated more thoroughly.

### Model evaluation

The R^2 ^value can be interpreted as "the amount of observed variation explained by the regression model". The mean R^2 ^values in table 2 show that all models generated values above 0.99. Obviously, differences in the 3^rd ^decimal place of R^2 ^are not a good measure of model performance, so the Altman-Bland method is more informative.

In the present study, the gold standard CT method has an intra-assay variation (error) of 24%, which is close to previously reported values [15]. This error is a sum of the inaccuracies in fluorescence measurement, thermocycling, pre-PCR procedures, and the CT fractional cycle estimate. Most of these inaccuracies are common to both calculation methods. In NLR, 4 or 5 variables are estimated in each analysis (C_1/2_, F_max_, k, F_b_, *f*), and each of these estimates contain intrinsic error. Thus, the resulting intra-assay variation is a combination of inaccuracies in the pre-PCR procedures, equipment errors, and errors in the variable estimates. Thus, in effect at least 35% of the total 59% error in model 4 is generated by the mathematical model itself.

Of the four different modifications to the original model tested, changes in R^2 ^were minute – but in terms of error all modifications tested had a negative impact on the model, probably due to the increased number of variables estimated. Model 4 (log_10 _transformation of raw data) produced marginally lower bias and marginally higher error, and this is the only modification that was not directly harmful to model performance.

The high-performance assays used in this study are an optimal setting for CT analysis, and our evaluation may therefore be quite conservative in terms of demonstrating the advantages of NLR. In assays with varying PCR efficiency, the NLR method may yet prove to be more precise than the CT method. This, however, awaits systematic evaluation.

### Conversion factor

The use of an absolute conversion factor, or optical calibration, has been evaluated previously in different analysis models [6,13]. The three data clouds in figure 5 were generated with separate conversion factors, and their distribution shows a pattern of random variation, underlining that our conversion factor assessment was inaccurate. However, the conversion factor only affects the absolute sample value and not the intra-assay variation, nor the rank position of a sample in the data set.

Probe-based chemistry theoretically offers a stoichiometric calibration, as each probe has one reporter and one quencher molecule. In effect, the conversion factor should be universal and independent on the template measured. The conversion factors calculated in this series ranged × 3.9 from lowest to highest, and this also indicates that the precision of our conversion factors was less than optimal. Stoichiometric calibration was investigated in detail by Swillens et al [13], who lowered probe concentration to define probe as the limiting factor of fluorescence accumulation. This approach assumes a precise probe concentration and a 100% conjugation and purity. As the problem of signal to noise ratio is inherent in all probe-based assays, a reduction of probe concentration lowers the detection window even further and may impair precision.

### Alternative mathematical models

For curve smoothing combined with the CT method, the sigmoidal curve fit may not be optimal – as the Gompertz function [16] shows a better fit both with the steep increase phase and the late plateau phase. The Gompertz algorithm is not suitable for estimation of initial fluorescence, though (tested, not shown).

To calculate the initial copy number accurately, the efficiency of each cycle must be estimated:

N0=NCTE0×E1×…×ECT−1
 MathType@MTEF@5@5@+=feaafiart1ev1aaatCvAUfKttLearuWrP9MDH5MBPbIqV92AaeXatLxBI9gBaebbnrfifHhDYfgasaacH8akY=wiFfYdH8Gipec8Eeeu0xXdbba9frFj0=OqFfea0dXdd9vqai=hGuQ8kuc9pgc9s8qqaq=dirpe0xb9q8qiLsFr0=vr0=vr0dc8meaabaqaciaacaGaaeqabaqabeGadaaakeaacqWGobGtdaWgaaWcbaGaeGimaadabeaakiabg2da9maalaaabaGaemOta40aaSbaaSqaaiabdoeadjabdsfaubqabaaakeaacqWGfbqrdaWgaaWcbaGaeGimaadabeaakiabgEna0kabdweafnaaBaaaleaacqaIXaqmaeqaaOGaey41aqRaeSOjGSKaey41aqRaemyrau0aaSbaaSqaaiabdoeadjabdsfaujabgkHiTiabigdaXaqabaaaaaaa@44D9@

In theory each of these efficiencies could be measured directly on the fluorescence curve. In practice, however, only a few points on the PCR curve yield workable efficiency estimates because the early plateau phase is dominated by background noise. Rutledge recently proposed an alternative model for estimation of maximal efficiency based on the sigmoidal model [17]. As the efficiency is directly calculable in the log-linear phase [4], the important extremes of efficiency (E_0 _and E_CT_) can be assessed. Further work will show if this model is workable, or if it will fall short on the problem of multiple estimates.

## Conclusion

NLR is automatable and may be a powerful tool for analysis of fluorescence data from real-time PCR experiments. The unfavourable signal to noise ratio of the probe-based assays did not impair NLR analysis. The versatility of NLR depends on the precision needed – but if adaptable, this analysis method may save both time and resources in the laboratory. Further work is needed as to improve precision of the fluorescence-copy number conversion factor in order to reduce the bias observed in this study.

It is indeed possible to obtain absolute quantitation from real-time data without a standard curve. In an optimised assay, however, the CT method remains the gold standard due to the inherent errors of the multiple estimates used in NLR.

## Methods

### RNA extraction

Forty-four biopsies of human gastric mucosa, collected by endoscopy of outpatients referred for dyspepsia, were included in this study after written informed consent. Biopsies were stored in *RNA-Later *(Ambion, Austin, Texas, USA) until extraction by the *Trizol *method (Invitrogen, Carlsbad, California, USA) according to the manufacturer's instructions. A standardised amount of total RNA (1μg) was reverse transcribed by *Superscript II *(Invitrogen), and cDNA was stored at -70°C. Samples were measured in duplicate by real-time PCR in an ABI-Prism 7900 instrument using *TaqMan *chemistry and SDS 2.1 software (Applied Biosystems, Foster City, California, USA), and a standard protocol in 25μL format. Three different templates were measured; table 1 shows the primers and probes, manufactured as custom oligos by Eurogentec, Seraing, Belgium. The absolute standard was produced by serial dilution of a dsDNA PCR product, purified by gel band analysis/extraction (GFX columns, Amersham, Piscataway, NJ, USA), sequenced (BigDye 2.0, Applied Biosystems) and quantified by spectrophotometry (Eppendorf Biophotometer, Hamburg, Germany). Based on repeated standard curves, all three assays performed well with calculated mean efficiencies above 1.99, and standards with concentrations of 100 copies/μL or more yielded CT values with a narrow 95%CI. At lower concentrations (10 and 1 copies/μL) CT values showed increasing standard error, compatible with increasing stochastic effects at low concentrations. The assays chosen have different expression levels in the tissue analysed (ACTB>IL18>TNFA). Raw fluorescence readings were exported from SDS as "clipped" text files which are readable by the statistics software. Regression analysis was performed in SPSS 12.0.1 (SPSS Inc., Chicago, Illinois, USA).

### Regression models

The primary regression model used for curve fitting has been published previously [5,6]:

Equation 1: FC=Fmax⁡1+e−(C−C1/2k)+Fb
 MathType@MTEF@5@5@+=feaafiart1ev1aaatCvAUfKttLearuWrP9MDH5MBPbIqV92AaeXatLxBI9gBaebbnrfifHhDYfgasaacH8akY=wiFfYdH8Gipec8Eeeu0xXdbba9frFj0=OqFfea0dXdd9vqai=hGuQ8kuc9pgc9s8qqaq=dirpe0xb9q8qiLsFr0=vr0=vr0dc8meaabaqaciaacaGaaeqabaqabeGadaaakeaacqWGgbGrdaWgaaWcbaGaem4qameabeaakiabg2da9maalaaabaGaemOray0aaSbaaSqaaiGbc2gaTjabcggaHjabcIha4bqabaaakeaacqaIXaqmcqGHRaWkcqWGLbqzdaahaaWcbeqaaiabgkHiTmaabmaabaWaaSaaaeaacqWGdbWqcqGHsislcqWGdbWqdaWgaaadbaGaeGymaeJaei4la8IaeGOmaidabeaaaSqaaiabdUgaRbaaaiaawIcacaGLPaaaaaaaaOGaey4kaSIaemOray0aaSbaaSqaaiabdkgaIbqabaaaaa@4649@ Where *F*_*C *_is fluorescence at cycle *C*; *F*_*max *_is the maximal fluorescence intensity; *C *is cycle number; *C*_1/2 _is the fractional cycle at half of maximal fluorescence; *k *is a slope constant related to PCR efficiency; and *F*_*b *_is the background fluorescence.

This equation was tested with combinations of additional mathematical modifications to increase goodness-of-fit (R^2 ^closer to 1), as described below. For an overview of the 11 regression models, see table 2.

Baseline drift correction

In most of the reactions a slight, but significant linear increase of background fluorescence was observed. This baseline drift could be corrected by the introduction of a linear term in the regression model:

Equation 2: FC=Fmax⁡1+e−(C−C1/2k)+Fb+C×f
 MathType@MTEF@5@5@+=feaafiart1ev1aaatCvAUfKttLearuWrP9MDH5MBPbIqV92AaeXatLxBI9gBaebbnrfifHhDYfgasaacH8akY=wiFfYdH8Gipec8Eeeu0xXdbba9frFj0=OqFfea0dXdd9vqai=hGuQ8kuc9pgc9s8qqaq=dirpe0xb9q8qiLsFr0=vr0=vr0dc8meaabaqaciaacaGaaeqabaqabeGadaaakeaacqWGgbGrdaWgaaWcbaGaem4qameabeaakiabg2da9maalaaabaGaemOray0aaSbaaSqaaiGbc2gaTjabcggaHjabcIha4bqabaaakeaacqaIXaqmcqGHRaWkcqWGLbqzdaahaaWcbeqaaiabgkHiTmaabmaabaWaaSaaaeaacqWGdbWqcqGHsislcqWGdbWqdaWgaaadbaGaeGymaeJaei4la8IaeGOmaidabeaaaSqaaiabdUgaRbaaaiaawIcacaGLPaaaaaaaaOGaey4kaSIaemOray0aaSbaaSqaaiabdkgaIbqabaGccqGHRaWkcqWGdbWqcqGHxdaTcqWGMbGzaaa@4BB0@ where *f *is a constant.

#### Weighted analysis

In the late plateau phase, a deviation from the sigmoid pattern can be observed with SYBR green chemistry, and unfortunately this tendency seems to be even stronger with TaqMan chemistry. Rutledge addressed this problem by removing such values from the calculation [6]. We preferred a weighted regression – allowing for increase/decrease of impact of data – rather than removing values completely from the calculations. To automate this process, a "weight function" was devised based on a *C*_1/2 _estimate. This function generates a set of weights that is tailored to each specific reaction.

Equation 3: Weight=0.24+0.761+e−(C−C1/2−174.5)−0.951+e−(C−C1/21.8)+21+e−(C1/2−403)
 MathType@MTEF@5@5@+=feaafiart1ev1aaatCvAUfKttLearuWrP9MDH5MBPbIqV92AaeXatLxBI9gBaebbnrfifHhDYfgasaacH8akY=wiFfYdH8Gipec8Eeeu0xXdbba9frFj0=OqFfea0dXdd9vqai=hGuQ8kuc9pgc9s8qqaq=dirpe0xb9q8qiLsFr0=vr0=vr0dc8meaabaqaciaacaGaaeqabaqabeGadaaakeaacqWGxbWvcqWGLbqzcqWGPbqAcqWGNbWzcqWGObaAcqWG0baDcqGH9aqpcqaIWaamcqGGUaGlcqaIYaGmcqaI0aancqGHRaWkdaWcaaqaaiabicdaWiabc6caUiabiEda3iabiAda2aqaaiabigdaXiabgUcaRiabdwgaLnaaCaaaleqabaGaeyOeI0YaaeWaaeaadaWcaaqaaiabdoeadjabgkHiTiabdoeadnaaBaaameaacqaIXaqmcqGGVaWlcqaIYaGmaeqaaSGaeyOeI0IaeGymaeJaeG4naCdabaGaeGinaqJaeiOla4IaeGynaudaaaGaayjkaiaawMcaaaaaaaGccqGHsisldaWcaaqaaiabicdaWiabc6caUiabiMda5iabiwda1aqaaiabigdaXiabgUcaRiabdwgaLnaaCaaaleqabaGaeyOeI0YaaeWaaeaadaWcaaqaaiabdoeadjabgkHiTiabdoeadnaaBaaameaacqaIXaqmcqGGVaWlcqaIYaGmaeqaaaWcbaGaeGymaeJaeiOla4IaeGioaGdaaaGaayjkaiaawMcaaaaaaaGccqGHRaWkdaWcaaqaaiabikdaYaqaaiabigdaXiabgUcaRiabdwgaLnaaCaaaleqabaGaeyOeI0YaaeWaaeaadaWcaaqaaiabdoeadnaaBaaameaacqaIXaqmcqGGVaWlcqaIYaGmaeqaaSGaeyOeI0IaeGinaqJaeGimaadabaGaeG4mamdaaaGaayjkaiaawMcaaaaaaaaaaa@72DA@

The constant initialises the weight at a base level; the second term gradually increases the weight from around 20 cycles before *C*_1/2. _The third term decreases the weight rapidly at *C*_1/2, _and the fourth term reduces the impact of the weights at *C*_1/2 _above 35.

#### Log10 transformation

An alternative way of dealing with late plateau phase drift is log_10 _transformation of fluorescence data, which changes the profile of the fluorescence curve to a more sigmoid pattern. In log_10 _transformed fluorescence data, however, the background fluorescence makes the early plateau phase very noisy – so a second, similar weight profile algorithm was devised to lessen the impact of early plateau phase data on the calculations.

#### Backgr. correction

The basis of CT analysis is fluorescence data corrected for Backgr. detection (background noise). When exporting data from SDS, two tables are generated, one with raw data (no correction), and one with background subtraction (Backgr. corrected).

#### Absolute quantitation

Calculation of the template-related initial fluorescence was made by substitution of C by 0:

Equation 4: F0=Fmax⁡1+e(C1/2k)
 MathType@MTEF@5@5@+=feaafiart1ev1aaatCvAUfKttLearuWrP9MDH5MBPbIqV92AaeXatLxBI9gBaebbnrfifHhDYfgasaacH8akY=wiFfYdH8Gipec8Eeeu0xXdbba9frFj0=OqFfea0dXdd9vqai=hGuQ8kuc9pgc9s8qqaq=dirpe0xb9q8qiLsFr0=vr0=vr0dc8meaabaqaciaacaGaaeqabaqabeGadaaakeaacqWGgbGrdaWgaaWcbaGaeGimaadabeaakiabg2da9maalaaabaGaemOray0aaSbaaSqaaiGbc2gaTjabcggaHjabcIha4bqabaaakeaacqaIXaqmcqGHRaWkcqWGLbqzdaahaaWcbeqaamaabmaabaWaaSaaaeaacqWGdbWqdaWgaaadbaGaeGymaeJaei4la8IaeGOmaidabeaaaSqaaiabdUgaRbaaaiaawIcacaGLPaaaaaaaaaaa@3FC5@

The "optical calibration" was performed by running NLR on the reactions with known copy number (the absolute standard), and a conversion factor *CF *was calculated from the estimated *F*_0_.

Equation 5: CF=CopynumberF0
 MathType@MTEF@5@5@+=feaafiart1ev1aaatCvAUfKttLearuWrP9MDH5MBPbIqV92AaeXatLxBI9gBaebbnrfifHhDYfgasaacH8akY=wiFfYdH8Gipec8Eeeu0xXdbba9frFj0=OqFfea0dXdd9vqai=hGuQ8kuc9pgc9s8qqaq=dirpe0xb9q8qiLsFr0=vr0=vr0dc8meaabaqaciaacaGaaeqabaqabeGadaaakeaacqWGdbWqcqWGgbGrcqGH9aqpdaWcaaqaaiabdoeadjabd+gaVjabdchaWjabdMha5jabd6gaUjabdwha1jabd2gaTjabdkgaIjabdwgaLjabdkhaYbqaaiabdAeagnaaBaaaleaacqaIWaamaeqaaaaaaaa@3FB7@

The regression models were written in SPSS syntax. On a decent PC (2.6 GHz P4, 256 MB RAM, XP pro), the algorithm processes an entire 96 reaction plate in less than 2 minutes.

### Evaluation of output data

The three SDS files were subjected to analysis by CT/standard curve and by all 11 NLR algorithms. For each model, a mean R^2 ^was calculated for comparison of goodness of fit between models. The data sets were then subjected to Altman-Bland analysis [18]. Two types of Altman-Bland plots were generated. In the first type, intra-assay variability was evaluated in plots of *numerical *difference between duplicate values (termed "error") vs. mean of the duplicate values. If the error is independent of mean value, the 95^th ^percentile is a measure of the overall intra-assay variability. If independence is not observed (i.e. patterns are observed in the scatter plot), appropriate transformation of raw data (here: the calculated copy numbers) or partitioned analysis must be applied before the error can be evaluated. The second type of Altman-Bland plot was aimed at evaluation of inter-method agreement (i.e., comparison of NLR vs. CT derived values). Plots of the point-to-point differences (termed "bias") versus the means of results derived by the two methods were inspected and rules of independence applied. The mean of the observed bias values yields a reasonable measure of the systematic error of estimates.

### Ethics

The project was recommended by the regional committee of medical research ethics (REK Northern Norway), ref # 200100973-5/IAY/400.

## Authors' contributions

RGO conceived of the study, designed the project, and performed the experiments and calculations. All authors contributed in discussion of regression models, conclusions, and in preparation of this paper.

## Additional files

All files include 4 sections: A: Imports of raw data into an SPSS data file. B: Performs nonlinear regression on absolute standards for calculation of calibration factor. C: Performs nonlinear regression on all remaining reaction traces. C: Collects data in an SPSS data file and calculates absolute copy numbers for each reaction.

## Supplementary Material

Additional File 1 The original sigmoid model with no modifications, performed on data that were not corrected for background fluorescence. Click here for file

Additional File 2 As in model 1 but corrected for baseline drift by a linear term in the regression model. Click here for file

Additional File 3 As in model 2 but the raw data was weighted with impact on the early plateau phase. Click here for file

Additional File 4 As in model 1 but the raw data was transformed with a log_10 _before NLR. Click here for file

Additional File 5 As in model 4 with addition of baseline drift correction. Click here for file

Additional File 6 As in model 5 but the raw data was weighted with impact on the early plateau phase. Click here for file

Additional File 7 As in model 6 but the raw data was weighted with impact on the late plateau phase. Click here for file

Additional File 8 Raw data corrected for background fluorescence, weight emphasis on the early plateau phase. Click here for file

Additional File 9 Raw data corrected for background fluorescence, log_10 _transformed, and weighted with emphasis on late plateau phase. Click here for file

Additional File 10 Raw data corrected for background fluorescence. Click here for file

Additional File 11 As in model 10 but the raw data was transformed by log_10_. Click here for file

## Figures and Tables

**Table 1:** The primer and probe sequences used in TaqMan PCR

Assay	Primer	Sequence
ACTB		PubMed accession number: BC002409
	Forward	5' TGCCGACAGGATGCAGAAG 3'
	Reverse	5' GCCGATCCACACGGAGTACT 3'
	Probe	FAM 5' AGATCAAGATCATTGCTCCTCCTGAGCGC 3' TAMRA

TNFA		PubMed accession number: X01394
	
	Forward	5' CACGCTCTTCTGCCTGCTG 3'
	Reverse	5' GATGATCTGACTGCCTGGGC 3'
	Probe	FAM 5' CCAGAGGGAAGAGTTCCCCAGGGAC 3' TAMRA

IL18		PubMed accession number: BC007461
	
	Forward	5' ATCGCTTCCTCTCGCAACA 3'
	Reverse	5' CATTGCCACAAAGTTGATGCA 3'
	Probe	FAM 5' CAGGAATAAAGATGGCTGCTGAACCAG 3' TAMRA

Primers and probes were generated by Primer Express 1.0 using sequence data from Pubmed. All assays cross exon splicing points in order to avoid detection of genomic DNA. Following assay design, specificity was double-checked by a BLAST search on the NCBI website.

**Table 2:** An overview of regression models and key performance measures

Regression Model					Results			
	Backgr. Corr.	Log_10 _transform	Weight	Baseline drift corr.	Bad fits	Mean R^2^	Intra-assay variation	Numerical Bias

1	No	No	No	No	No	0.9987	58 %	89 %
2	No	No	No	Yes	No	0.9988	83 %	102 %
3	No	No	←	Yes	No	0.9982	80 %	121 %
4	No	Yes	No	No	No	0.9987	59 %	85 %
5	No	Yes	No	Yes	No	0.9991	94 %	97 %
6	No	Yes	←	Yes	Yes	0.9986	104 %	130 %
7	No	Yes	→	Yes	No	0.9985	101 %	115 %
8	Yes	No	←	No	No	0.9978	94 %	166 %
9	Yes	Yes	→	No	Yes	0.9768	29963 %	340 %
10	Yes	No	No	No	No	0.9922	83 %	102 %
11	Yes	Yes	No	No	Yes	0.9607	36336 %	526 %
CT	Yes						24 %	

An overview of the 11 regression models evaluated. In the left part of the table, the modifications of each model are stated. In the right part of the table, key features of the analysis are shown. Arrows in the weight column indicate early plateau phase (←) and late plateau phase (→).

**Table 3:** The impact of mathematical modifications on model performance

Model change	Mean R^2 ^change	Intra-assay variation	Bias
Backgr. corrected data	-0.0065	+27%	+15%
Log_10 _transformation	0	+1%	-4%
Correction of baseline drift (linear)	+0.0001	+20%	-7%
Correction of baseline drift (Log_10_)	+0.0004	+35%	+12%
Weight early cycles (RAW)	-0.0006	+3%	+40%
Weight early cycles (Backgr. corrected)	+0.0056	+11%	+64%
Weight late cycles	-0.0006	+7%	+18%

Performance of model modifications applied to different regression models, evaluated as single modifications. Even though four modifications show better or unchanged mean R^2^, all modifications actually add to the intra-assay variability.
